# Isolated Polyethylene Exchange in Revision Total Knee Arthroplasty: A Review of Indications and Outcomes

**DOI:** 10.3390/jcm14196779

**Published:** 2025-09-25

**Authors:** Alex M. Moses, Michaela E. Cushing, Mason A. Fawcett, Nicolas Dohse, Obinna O. Adigweme, Cameron K. Ledford

**Affiliations:** 1 UF Health Jacksonville, Jacksonville, FL 32209, USAmason.fawcett@jax.ufl.edu (M.A.F.); nicolas.dohse@jax.ufl.edu (N.D.); 2 Orlando Orthopaedic Center, Orlando, FL 32806, USA; obinna.adigweme@rothmanortho.com; 3 Mayo Clinic Jacksonville, Jacksonville, FL 32224, USA

**Keywords:** revision total knee replacement, stiffness, arthrofibrosis, prosthetic knee instability, periprosthetic joint infection, total knee replacement

## Abstract

**Introduction:** The use of modular components provides several advantages in total knee arthroplasty (TKA), including exchange of the polyethylene insert while retaining the stable components in the revision TKA. Compared to full, non-modular component revision TKA, isolated polyethylene exchange (IPE) has the advantage of decreased morbidity, faster rehabilitation, and acceptable outcomes. **Methods:** A review of published literature on revision TKA was conducted, with a specific focus on studies evaluating the use of IPE for managing complications such as stiffness, instability, and periprosthetic joint infection (PJI). **Results:** IPE with downsizing may be considered for patients with mild stiffness and stable, well-positioned implants that have increased polyethylene thickness, though expectations for motion gain should be cautious. There is no clear consensus on IPE for instability. Some studies report high re-revision rates, while others show clinical and functional improvement when the TKA is well-aligned, well-fixed, and intraoperative gap balance is achieved. Additionally, irrigation and debridement with polyethylene exchange (IDPE) may be effective for acute TKA PJI management, particularly within the first two weeks of symptom onset. **Conclusions:** Based on current literature, IPE in aseptic TKA revisions may be effective for stiffness or instability when implants are well-fixed and well-aligned—particularly if polyethylene constraint can be adjusted for instability or downsized for stiffness. The role of IDPE in acute TKA PJI is better defined in the literature, with strong emphasis on its time-sensitive effectiveness—most notably within the first two weeks of symptom onset.

## 1. Introduction

Total knee arthroplasty (TKA) is a safe and effective treatment for debilitating osteoarthritis, demonstrating excellent long-term survivorship [1]. It is estimated that 700,000 knee replacement procedures are performed annually in the United States [2]. Unfortunately, the burden of revision TKA is increasing substantially, with over 337,000 revisions occurring from 2009 to 2013 [3]. Isolated tibial component polyethylene exchange (IPE) has become a well-used revision option. Its modular component design provides several advantages, including the option to exchange the polyethylene (PE) insert while retaining the well-fixed components in the revision setting. This is an attractive option compared to complete component revision due to decreased operative time, preserved bone stock, and shorter hospital stays, amongst other factors; however, it has not come without its learning curve and complications [4].

Tibial components have evolved, with early components typically being Monoblock All-PE designs, which provided good initial fixation but limited intraoperative flexibility [5]. The 1980s saw the development and widespread adoption of modular tibial trays, composed of a metal baseplate and a separate PE insert. This modularity allowed for the customization of insert thickness, geometry, and degree of constraint during surgery to better accommodate patient anatomy. Modular tibial components also provided an opportunity for simple PE liner exchange as opposed to the complete tibial component for osteolysis in the revision setting [6]. However, this design introduced a new challenge of backside wear as the backside interface between the insert and metal tray became susceptible to micromotion, leading to the generation of PE debris. This contributed to osteolysis and implant loosening over time [7,8].

This finding has led to extensive research directed towards minimizing PE wear. PE manufacturing for total knee arthroplasty began with the use of conventional ultra-high-molecular-weight polyethylene (UHMWPE). However, it was later found to be susceptible to oxidative degradation, wear, and subsequent osteolysis, which led to implant failure [9]. In the late 1990s and early 2000s, highly crosslinked polyethylene (HXLPE) was developed by using higher irradiation doses and thermal treatments to improve wear resistance, though initial formulations raised concerns about reduced mechanical toughness and increased risk of fracture in total knee arthroplasty applications [10]. More recent advances have focused on second-generation HXLPE, including the incorporation of antioxidants such as vitamin E and sequential annealing, which aim to further reduce wear and oxidation while maintaining mechanical integrity, and these materials are now increasingly used in clinical practice for total knee arthroplasty [11].

For this reason, advancements in PE bearing materials have been aimed at improving durability and decreasing the number of wear particles to reduce the incidence of particle-induced osteolysis [12]. HXLPE was introduced with the goal to address oxidation and wear issues. Contemporary HXLPE is either crosslinked via gamma or electron beam radiation and further stabilized by thermal annealing or remelting. Annealing involves heating below the crystalline melting point, preserving strength but leaving residual free radicals that can oxidize in vivo. Conversely, remelting heats above the melting point, eliminating free radicals at the expense of reducing crystallinity and compromising mechanical properties [12,13]. To combat the formation of these free radicals, the biological antioxidant, vitamin E, can be added to these polymers either before or after gamma irradiation. The addition of vitamin E prior to irradiation does adversely decrease the extent of cross-linking that occurs, but it allows for higher levels of vitamin E in the polymer compared with post-irradiation addition [11].

Manufacturing techniques also play a key role, with direct compression molding (DCM) of the PE creating liners that are more uniform and wear-resistant compared to machine inserts, resulting in less backside wear with the more desirable modular designs [8]. The way that the PE resin is manufactured also has clinical implications. A 2021 retrieval analysis found that liners created via ram extrusion with added calcium stearate demonstrated shorter service life and inferior wear rates compared with net-shape molded inserts without calcium stearate [14]. The packaging of these components is also vital to their longevity and wear rates, with oxygen-impermeable vacuum-seal bags being essential for component preservation [15].

Mid-term clinical studies have shown that HXLPE is safe and effective in TKA, with significant reductions in wear rates and osteolysis without the increase in mechanical failures when compared to conventional PE inserts [12]. Despite these advancements in material properties and manufacturing, PE wear depends on many different factors. Implant design, surgical technique, patient anatomy and activity level all contribute to failure. Importantly, younger, more active patients are particularly susceptible to osteolysis resulting from accelerated PE wear, highlighting critical considerations concerning revision risk and overall TKA longevity [7,12]. PE wear is one of the most significant contributing factors to mid- and long-term clinical outcomes in TKA, highlighting the importance of further advancements, as well as revision options, which decrease morbidity. Despite the discrepancy in wear rates, data from large joint replacement registries show mixed findings when comparing conventional UHMWPE to HXLPE. The Australian registry reported a lower 10-year revision rate with HXLPE, particularly in younger patients, while the UK registry found lower revision rates with conventional UHMWPE at up to 12 years. In contrast, U.S. registry data showed no meaningful difference in revision outcomes between the two materials, regardless of antioxidant use [11].

Overall, PE remains a reliable bearing surface, and its use in modular implants is nearly universal. Exchange of a PE insert is widely accepted for PE wear or fracture in the absence of malalignment or loosening, but its role in non-PE–related complications remains debated. This review focuses on stiffness, instability, and infection, as they are among the most frequent and clinically significant causes of revision TKA. In addition, we address PE wear/osteolysis and patellar component failure as important scenarios in which IPE may also be applicable [3].

## 2. Methods

A narrative review of published work on revision TKA and the use of IPE was conducted. A PubMed database search was performed to find relevant studies up 4 June 2025, using terms which consisted of Medical Subject Headings (MeSH) and subheadings, text words, and word variations for ‘polyethylene’; ‘polyethylene wear’; ‘osteolysis’; ‘total knee arthroplasty’; ‘stiffness’; ‘instability’; ‘infection’; ‘patellar component’; ‘polyethylene exchange’; ‘manufacturing’. Peer-reviewed clinical studies, including case series, cohort studies, and clinical trials, were considered eligible if they reported outcomes of IPE performed for complications such as wear, osteolysis, stiffness, instability, or infection. In addition to the above criteria, studies were selected based on their methodological quality, clinical relevance, and their representation of prevailing treatment paradigms at the time of publication. This approach was intended to ensure that the review reflects both historical context and contemporary practice. Excluded in the study were conference abstracts, personal correspondence, non-clinical/biomechanical reports, and animal studies. No restrictions on language, date, or publication status were applied. 

## 3. Results

### 3.1. Polyethylene Wear and Osteolysis 

Since the advent of polyethylene (PE) liners in the 1960s, PE wear has been a persistent concern in TKA. Wear reportedly occurs from a combination of rolling, sliding and rotational movement between the bearing surfaces, which ultimately causes delamination, pitting and fatigue failure [16]. This wear is problematic for two primary reasons: mechanical dysfunction and osteolysis.

As wear progresses, it can alter flexion and/or extension gaps, potentially leading to late instability. While wear is becoming less common due to improvement in PE manufacturing [17], it was still found to be implicated in TKA failure mechanisms in 2% of acute TKA revisions and 9% of late TKA revisions as of 2014 [18]. In severe cases, the femoral condyle may begin articulating directly with the tibial baseplate, resulting in metallosis and catastrophic implant failure [19]. Additionally, PE wear generates particulate debris that triggers a macrophage-mediated inflammatory response (osteolysis). This can lead to bone resorption around the implant, ultimately causing aseptic loosening or fracture [16].

Historically, due to the high prevalence of wear, surgeons closely monitored their arthroplasty patients for early signs of osteolysis or wear. However, revision surgery carries its own risks, and determining the optimal timing for intervention has always been a subject of debate. In the setting of wear, the literature supports early IPE as intervention before the onset of severe wear is associated with better outcomes [4,20]. Additionally, direct comparisons to single and full component exchange, if components are not loose, show equal outcomes [21]. However, it should be noted that IPE in the setting of severe wear is at increased risk of re-revision and subsequent component exchange [4]. If osteolysis is present, one should consider advanced imaging to better evaluate the risk of structural compromise [22]. Small lesions are often observed closely without intervention, especially if the patient is medically unfit for surgery [22]. If lesions become progressive, large, symptomatic, or associated with component loosening, one should consider intervention and bone grafting [22]. If components are stable, IPE can be performed in addition to bone grafting of lesions [20]. However, if components are loose, full component revision should be pursued [20].

As previously mentioned, IPE can be effective in the setting of osteolysis and wear without component loosening, but one should consider special circumstances. When the tibial component is nonmodular, IPE is not an option, and full component revision is the only option. At the turn of the century, highly cross-linked polyethylene was introduced, marking a significant advancement. Numerous studies have demonstrated its lower wear rates and reduced need for revision [23,24]. A 2021 retrieval study of 1585 modular tibial inserts found that highly crosslinked polyethylene was associated with lower wear rates when controlling for both patient and implant variables [25]. If wear is noted on newer-generation PE liners, the treating surgeons should critically evaluate both the track record of that specific polyethylene liner and component positioning that may be the underlying reason for early failure [22]. In summary, early wear and osteolysis can be observed. However, if IPE can be performed before late instability, component failure, or osteolysis progression, outcomes tend to be improved.

### 3.2. Stiffness

Stiffness after TKA can be debilitating and occurs in 1–7% of TKAs [26,27]. Without adequate range of motion, patients are often unable to perform many activities of daily living, such as walking on level ground, rising from a chair, or descending stairs. Risk factors include limited preoperative range of motion, poor patient motivation, extended duration of immobilization, technical issues, low pain threshold, and reflex sympathetic dystrophy [28,29].

The treatment of stiffness after TKA is historically difficult. Initial management includes aggressive physiotherapy, adequate analgesia, and reduction in swelling. If stiffness persists within three months postoperatively, manipulation under anesthesia (MUA) should be considered. MUA has been shown to be safe and effective within three months [30]. After three months, options include progressive splinting, arthroscopic arthrolysis, open arthrolysis and polyethylene exchange, and component revision.

The indication for polyethylene exchange in treating stiffness after TKA has shown variable results in the literature (Table 1). Early reports by Babis et al. showed poor results of arthrolysis and isolated tibial insert downsizing on seven well-aligned, well-fixed knees for idiopathic stiffness after TKA [31]. Two of those patients required subsequent component revision, and the remaining 5 had persistent pain after a mean of 4.2 years. Mont et al. later described moderately better outcomes in their series of 18 stiff knees [32]. In this study, patients underwent IPE after arthrolysis as well as intensive postoperative physical therapy. The physiotherapy regimen included early and aggressive mobilization, functional bracing, and close supervision. The regimen involved frequent, supervised physical therapy sessions with a focus on active and passive range of motion exercises, strengthening, and functional training, with adjustments made based on individual progress. Functional bracing was used to maintain gains in motion between therapy sessions. The average range of motion increased by 31 degrees with 67% good and excellent results based on Knee Society objective scores. However, the authors noted that a significant factor contributing to the successful outcomes may have been their intensive postoperative physical therapy protocol.

Keeney et al. reported more predictable clinical and functional outcome improvements when soft tissue releases and tibial insert downsizing were used in comparison to complete component revision for patients with postoperative stiffness [33]. Patients who underwent IPE had improvements in knee motion arc, mean clinical scores, and mean functional scores. In contrast, patients with component revision had a lower mean improvement in knee motion arc and little change in clinical or functional scores. It should be noted that the patients in the complete revision cohort had worse initial flexion contractures, while patients undergoing IPE had mild flexion contractures (less than 15 degrees). A later study by Hug et al. echoed the findings seen in the study by Keeney et al. They reported improved functional scores and a 32-degree improvement in the arc of motion when preoperative flexion contracture averaged 17 degrees [34].

More recent studies indicate that full component revision generally results in superior functional outcomes and range of motion compared to IPE for arthrofibrosis or stiffness after TKA, though IPE may be considered in select cases with well-fixed, well-aligned components and may yield lower re-revision rates in cases of idiopathic stiffness. A comparative study by Dubin et al. found that IPE resulted in inferior outcomes when compared to full component revision, with lower mean final flexion (92 degrees vs. 102 degrees), lower Knee Injury and Osteoarthritis Outcome Scores for Joint Replacement (KOOS, JR) and higher re-revision rates [35]. Xiong et al. specifically evaluated treatment for idiopathic versus non-idiopathic stiffness. They reported lower re-revision rates and similar ROM (maximum flexion 96.8 degrees vs. 88.4 degrees, *p* = 0.06, and flexion ROM 93 degrees vs. 83.9 degrees, *p* = 0.07) in patients treated with isolated tibial insert exchange compared to full component revision for idiopathic stiffness. However, for cases of non-idiopathic stiffness, they reported higher re-revision rates with IPE (50% vs. 27.5%). These observed differences could be due to earlier initiation of rehabilitation, which may be effective for treating idiopathic stiffness as opposed to non-idiopathic cases such as implant malposition or aseptic loosening [35,36].

In review of the literature, IPE may be a reasonable option for managing postoperative stiffness in select patients. Ideal candidates typically have well-fixed, well-positioned implants, increased polyethylene thickness allowing for downsizing, and mild stiffness (<10° flexion contracture and >90–100°). When performed beyond one year from the index procedure and following failed manipulation under anesthesia (ideally <3 months from TKA), this approach—combined with structured physiotherapy—offers a lower morbidity alternative to full component revision. However, patients should be counseled to maintain realistic expectations regarding range-of-motion gains, recalcitrant stiffness, and potential risk of flexion instability with excessive downsizing.

**Table 1:** Stiffness.

Author	Year	Sample Size	Intervention	Follow Up	Outcome
Babis et al. [31]	2001	7	Isolated tibial insert exchange and arthrolysis (for stiffness after TKA, with well-fixed/aligned components)	Mean 4.2 years (range 2–8 years)	Minimal improvement in arc of motion (mean increase from 38.6° to 58°); poor pain and function scores; 2 knees required re-revision; overall poor outcomes
Keeney et al. [33]	2005	25	Revision TKA for restricted motion: compared limited approach (soft tissue release, component retention, tibial insert downsizing) vs. full component revision	Mean 36.7 months	Limited approach: mean arc gain 25.7°, clinical score +37.8, functional score +20.8; Full revision: mean arc gain 17.9°, minimal clinical/functional score change; limited approach may be appropriate for select patients
Mont et al. [32]	2006	18	Surgical arthrolysis plus intensive, protocolized rehabilitation (including functional bracing)	Mean 30 months	Mean ROM gain of 31°; 94% had improved motion; 2/3 had good/excellent Knee Society scores; all improved clinically, but functional results less predictable
Hug et al. [34]	2018	69	Protocol-driven revision for stiffness: debridement + polyethylene exchange, single component revision, or full component revision	Mean 43 months (range 12–205 months)	Mean arc of motion improved from 53° to 87°; KSS knee score from 42 to 70; function score from 41 to 68; full revision yielded greatest improvements (arc +45°), but all approaches improved motion and function
Xiong et al. [36]	2021	189 (101 idiopathic stiffness, 88 non-idiopathic stiffness)	IPE (42) vs. Component revision (59)	Mean 4.4 years (idiopathic) mean 4.0 years (non-idiopathic)	IPE had lower re-revision rate (16.7% vs. 31%), similar ROM improvement for idiopathic stiffness; IPE had higher re-revision rates (50% vs. 27.5%) for non-idiopathic stiffness.
Dubin et al. [35]	2024	49	Full component revision vs. IPE for arthrofibrosis	Mean 3.8 years	Full revision superior: higher KOOS, JR (65 vs. 55), greater flexion (102 degrees vs. 92), similar MUA/LOA rates; IPE had higher re-revision rate (37.5% vs. 3%)

### 3.3. Instability

Instability is one of the most common causes of failure in TKA, accounting for about 11% of revision procedures from 2012 to 2019 [37]. Instability symptoms include pain, recurrent effusions, diffuse tenderness, and “giving-away” of the knee. The causes of instability include surgical error, implant design, and patient-related factors. Types of TKA instability are flexion, extension (symmetric and asymmetric), and genu recurvatum [38]. Flexion instability is generally caused by a loose flexion gap or PCL rupture/injury in cruciate-retaining implants. Over-resection of the distal femur or proximal tibia, leading to a failure to fill the space with components, causes symmetric extension instability. Asymmetric extension instability, which is much more common, is usually due to inadequate soft tissue balancing or intraoperative ligamentous injury. Genu recurvatum, often due to neuromuscular disorders, quadriceps weakness, or TKA dislocation, is rare. IPE is rarely a viable treatment option in this circumstance.

Studies on IPE for instability have historically emphasized unacceptably high failure rates when compared to component revision (Table 2). Babis et al. examined the effectiveness of IPE in revision TKA, finding a 44% failure rate at a mean three-year follow-up, with most re-revisions being due to recurrent instability [39]. Brooks et al. also reviewed the management of TKA instability [40]. Fourteen patients underwent IPE, mostly due to a flexion-extension mismatch. The authors found a 29% failure rate at a mean follow-up of 56 months. Similarly, Willson et al. reported a 29% rate of re-revision after IPE with a minimum 2-year follow-up (mean 5.6 years) [41]. It should be noted that none of the aforementioned studies consistently increased PE liner constraint or size.

A more recent retrospective study by Cooper et al. compared unstable TKAs managed with IPE with increasing insert constraint versus component revision [42]. A 19% failure rate was reported, which, contrary to previous literature, was similar to the failure rate in the component revision group. Knee Society function scores and arc of motion also had no statistically significant difference between component revision and IPE. The insert constraint was increased when the implant system allowed, which was about 4.4 mm on average. There was, however, a significantly higher re-revision rate when insert constraint was not increased at revision surgery. They concluded that IPE yields equivalent results when compared to component revision in properly selected cases and modern implant designs that allow increasing polyethylene constraint. Properly selected cases in this study are considered to be well-fixed and well-aligned components, with no evidence of infection, and instability that can be addressed by increasing the constraint of the polyethylene insert without revising the femoral or tibial components.

Moreover, Green and Haidukewych showed that flexion instability after primary TKA could be managed with IPE if component rotation, sizing, and fixation were excellent and the flexion and extension gaps could be balanced intraoperatively [43]. Well-balanced intraoperative gaps refer to the ability to achieve symmetric and stable flexion and extension gaps under stress testing, without excessive laxity or tightness, and with appropriate constraint adjustment when available. With this strict criterion, they showed an improvement in Knee Society pain and function scores and only a 6.5% re-revision rate after 41 months.

In contrast, a recent study by Tegethoff et al. advocates against IPE in TKA instability after their study showed a significantly increased risk of reoperation when compared to component revision (45% vs. 8.7%). Once again, increasing constraint was not performed routinely [44]. In 2024, Debbi et al. showed a 10% higher re-revision rate when comparing full component revision and IPE for instability. However, full component revision had higher rates of aseptic loosening, periprosthetic joint infection, and longer length of hospital stay [45].

Overall, a clear indication of IPE in TKA instability is still elusive. Studies with rigorous patient selection and use of modern modular implants with increased constraint report better outcomes for IPE, while studies with less selective criteria or without increased constraint show high failure rates. Additionally, while some studies indicate IPE results in an unacceptably high re-revision rate compared to complete component revision, one must realize that both patients and providers may be less willing to re-revise after complete component revision to reduce morbidity. Complications have been shown to be significantly increased in full component exchange [45]. In contrast, IPE is less morbid and bone preserving; thus, patients and providers may be more willing to re-revise if unsatisfied with the results. Considering all factors, IPE may be indicated in the well-aligned, well-fixed TKA if flexion and extension gaps are well-balanced intraoperatively and increased constraint is possible through modern design. If proper balancing cannot be obtained, one should consider complete component revision despite its higher morbidity, as IPE does seem to be at higher risk for reoperation. 

### 3.4. Periprosthetic Joint Infection

Periprosthetic joint infections (PJI) are among the most challenging and devastating problems following TKA. The incidence of PJI is 1–2% in primary TKAs, and it is one of the most common reasons for TKA revision [46,47]. Unfortunately, it is also associated with high mortality rates [48]. Along with the undesired effects on the patient, they also pose a significant economic burden. On average, the management of a single episode of PJI ranges from $44,000 to $158,000 [49]. Considering PJI’s prevalence, high cost, and increased mortality risk, effective treatment is of high priority.

Management of PJI is usually based on the duration of symptoms. Although there is some variation, acute infection is generally defined as being within 4–6 weeks from index TKA or at any time with symptom duration less than 4–6 weeks, whereas chronic infections are considered to be any infection lasting longer than 4–6 weeks. This time-dependent management strategy is heavily based on the bacteria’s ability to form biofilm. Biofilm provides a network of conduits that allow for bacterial communication and synergy. Moreover, not only does biofilm facilitate the cultivation of bacterial colonies on the prosthesis through symbiosis, but it also contributes to antibiotic resistance [50,51,52]. By creating physical barriers impenetrable to antibiotic agents and creating an environment that induces a slowed bacterial metabolic state, ultimately decreasing antibiotic uptake, biofilms have forced providers to pursue more invasive treatment options.

Due to biofilm formation, there is a consensus that the undesirable risks associated with 2-stage revision outweigh its effectiveness at eradicating infection [53,54]. Irrigation and debridement with component retention have resulted in unacceptably high failure rates in chronic infection. A large multicenter study found that 50% of the 216 cases of irrigation and debridement with isolated polyethylene exchange (IDPE) performed required re-revision in the setting of chronic TKA [55]. Additionally, Narayanan et al. demonstrated successful treatment in only 60% of TKAs undergoing IDPE [56]. Notably, the success rate was substantially greater in those treated within two weeks compared to those treated later than two weeks. Patients treated within two weeks of their symptom onset had a success rate of 82% (14/17) compared to 50% (19/38) in those treated beyond two weeks, demonstrating the importance of early infection detection and the limits of IDPE. Similarly, Son et al. evaluated patients with acute PJI treated with IDPE within five days of symptom onset [57]. IDPE in this scenario demonstrated excellent success rates of 88% (22/25), which the authors attributed to promptness between symptom onset and surgical intervention. For this reason, we will review the indications for IDPE in acute PJI.

Treatment for acute PJI is controversial. Options for management include arthroscopic debridement, open irrigation and debridement with or without polyethylene exchange, one-stage, or two-stage revision (Table 3). As mentioned above, two-stage revision is considered the gold standard for treating PJI. However, it is associated with significant postoperative morbidity and high cost. This makes IDPE appealing since it involves only a single operation, avoiding the morbidity associated with the two-stage revision. Historically, the literature regarding single-stage IDPE for acute PJI has yielded mixed results. Some of the variances in outcomes can be attributed to heterogeneity between study design, diagnostic criteria, surgical techniques, and perioperative management.

More recent studies have demonstrated improved infection control rates with better surgical techniques and improved knowledge of risk factors for failure. Zhang et al. support the importance of polyethylene exchange compared to isolated debridement [58]. They reviewed 25 TKAs with acute PJIs and found the success rate of debridement with polyethylene exchange was 61.11% (11/18), while patients who underwent debridement without polyethylene exchange had a 0% success rate (0/7). This study emphasized the value of polyethylene exchange and suggested that two-stage revision should be considered when implant designs are not amenable to insert exchange.

Unfortunately, even with insert exchange, many infections persist. To improve infection control after IDPE, several adjunctive techniques have been examined. Typically, IDPE consists of open synovectomy and debridement, lavage, and polyethylene exchange with retention of well-fixed components. Estes et al. examined the indication for two-staged debridement in managing acute PJI [59]. Their protocol consisted of initial debridement with retention of components and antibiotic bead insertion, followed by a second debridement within seven days, during which the beads were removed, and a new polyethylene component was inserted. With a minimum follow-up of 1.2 years, a 12.5% re-revision was observed. 10/20 patients were no longer on an antibiotic regimen, and 8/20 were on chronic suppression due to immunocompromised status. A follow-up study by Flierl et al. reported on the usage of antibiotic-impregnated calcium sulfate beads (27 knees, six hips) [60]. Theoretically, these beads are attractive due to their antibiotic elution and rapid absorption, which should avoid the need for re-operation. However, a 48% failure rate at an average of 1 year was reported leading the authors to conclude that calcium sulfate beads provided little benefit. Riesgo et al. examined a povidone-iodine lavage and vancomycin powder method [61]. In their protocol, after final polyethylene insertion, a dilute 0.35% povidone-iodine lavage was applied, followed by irrigation using pulsed lavage of 1 L sterile saline, and subsequently, 1 g of vancomycin powder was placed. The vancomycin-povidone iodine group experienced a significant reduction in failure rates (17%) at the final follow-up (minimum of 1 year) compared to the control group (37%).

Aside from surgical technique, non-technique-related factors have also been identified that increase the risk of IDPE failure. Methicillin-resistant S. aureus (MRSA) and methicillin-resistant coagulase-negative staphylococci (MRSE) are particularly difficult to treat with IDPE yielding an unacceptably low success rate in one study [62]. Similarly, Narayanan et al. reported a 68.66% success rate for all bacterium types and an 85.25% success rate after excluding MRSA and pseudomonas infections [56]. For this reason, some have suggested a two-stage exchange with acute PJI when virulent resistant organisms are present [63]. Other factors affecting outcomes include preoperative anemia, accompanying infections elsewhere in the body, preoperative CRP > 500 mg/L, and comorbidities such as chronic obstructive pulmonary disease and atrial fibrillation [64,65].

Overall, it does seem that there are indications for IDPE in TKA infection. Its efficacy is time-dependent and most effective during the first two weeks of symptoms. This is reflected in a recent review published in the New England Journal of Medicine, recommending that acute infections be managed with surgical debridement, antibiotics, and implant retention (DAIR), including IPE, provided there is no sinus tract, prosthesis loosening, or inability to close the wound [66]. Adjunctive procedures such as povidone-iodine lavage and vancomycin powder may improve infection eradication rates, although studies on this topic are sparse. It is essential for providers to recognize risk factors for failure, such as MRSA/MRSE infection and preoperative anemia, since previously failed IDPE is associated with increased failure rates for two-stage revision [65]. Lastly, it remains essential to individualize revision strategy, as emphasized in a recent Canadian population-based study from Wood et al., who demonstrated that tibial insert exchange and single-stage revision may be acceptable in frail or elderly patients who may not tolerate the morbidity of two-stage revision, despite the higher risk of revision for repeat infection [67].

### 3.5. Patellar Component

The patellofemoral articulation in total knee arthroplasty (TKA), often referred to as the “forgotten joint,” also includes a polyethylene (PE) component. The vast majority of cemented TKAs utilize an all-PE patellar design due to its lower risk of mechanical failure and fracture, and absence of backside wear, with 20-year survivorship rates exceeding 93% [68]. However, with the growing interest in press-fit TKA designs, metal-backed patellar components are becoming more common and have demonstrated survival rates higher than 95% up to 12 years post-op [69]. Cementless metal-backed patellar components were developed with the goal of providing long-term biological fixation, while simultaneously minimizing issues associated with all-PE components, including deformation, thermal damage, and third body cement wear. Meta-analyses have shown that cementless TKAs demonstrate comparable survival to cemented TKAs, with ten-year mean survival of 95.6% versus 95.3%, respectively, and similar 20-year survival rates of roughly 76% and 71% [70]. The rising incidence of TKAs performed annually, particularly on increasingly younger and larger patients, means patients are at higher risk for wear, aseptic loosening, and osteolysis with patellar components [70]. If these complications were to occur, these metal-backed patella components allow for greater modularity, making IPE of the patellar button more feasible [71].

Like the tibial PE component, the patellar button is susceptible to wear, loosening, and dysfunction over time. However, unlike the tibial PE—which is primarily affected by coronal and sagittal mechanical axis imbalances—the patellar component is more often compromised by axial malalignment, incorrect sizing, or improper positioning. These issues fall under the broader category of patellar maltracking. Patellar maltracking refers to the displacement of the patella from its normal central alignment, which may manifest as excessive patellar tilt, subluxation or complete dislocation [72]. Patellar maltracking can present in two forms: gross instability, which may lead to dislocation, and subtle instability, which often manifests as pain and functional impairment. These complications are typically caused by trauma, patient-specific factors or technical errors during component positioning, and treatment is based on the underlying etiology [72].

Surgical options utilized include multi-component revision, medial patellofemoral ligament (MPFL) reconstruction, tibial tubercle osteotomy, and lateral retinacular release [72]. IPE of the patellar button is generally not considered a valid treatment option if there is underlying axial malalignment. However, if the polyethylene liner is fractured or worn, outcomes after isolated patellar component in this situation have shown promising results, with one study showing only 1 out of 25 metal-backed patella components failing after 10 years [71].

When dealing with metal-backed patellar components, IPE is relatively straightforward. Whereas, all-PE patellar buttons are cemented, making removal more complex and often resulting in excessive bone loss (leaving < 8 mm of residual thickness). This is problematic, as extensor mechanism injuries in TKA can be catastrophic. If attempted IPE leads to such complications, alternative strategies may include patelloplasty, bone grafting, benign neglect, or, in rare cases, patellectomy.

Overall, IPE of the patellar button is feasible in select cases of metal-backed components with wear or fracture but is generally contraindicated in the setting of maltracking or when removal of all-polyethylene buttons risks excessive bone loss and extensor mechanism compromise.

## 4. Discussion

IPE is a bone-preserving, lower-morbidity option compared to one- or two-component revision. However, it seems evident based on the current assessment of the literature that the success of IPE is indication-specific and requires a deep understanding of complication etiology to decide whether IPE is a viable option for a particular patient. Overall, the evidence is heterogeneous, especially in regard to IPE in the setting of aseptic complication. Most of the studies were relatively small and retrospective in nature. By far, the best studied and predictable success of IPE is in the setting of acute prosthetic infection, in addition to thorough debridement and subsequent appropriate antibiotic treatment.

Wear and osteolysis can be effectively managed when performed early and components are stable. Bone grafting may be added for contained defects. However, one must recognize that increasing severity of osteolysis can cause component loosening, which will inevitably lead to catastrophic failure unless all loose components are revised. Regarding stiffness, the best outcomes after IPE were in the setting of small flexion contractures, well-fixed implants, and postoperative physiotherapy [32]. Of all aseptic indications, IPE in the setting of stiffness was the most unreliable [31]. Oftentimes stiffness is secondary to arthrofibrosis, not an oversized PE liner. A review of the literature reveals that an overstuffed TKA may benefit from IPE with downsizing of the PE inset [33]. However, lysis of adhesions and physical therapy, especially in more chronic and severe settings, are essential. Historically, IPE has been considered to be an inadequate treatment method for instability. However, with the advent of compatible PE liners with varying constraint, new data is now showing that increasing liner constraint can lead to near-equivalent outcomes as component revision if components are stable and extension and flexion gaps are balanced intraoperatively [42]. Additionally, IPE has been shown to be associated with few prosthetic fractures and infections when compared to full component revision [45].

As aforementioned, IPE in the setting of infection is by far the most well-defined. Timing is critical, with intervention less than 2 weeks from onset being paramount for efficacy. There is no role for IPE in chronic infections [53]. Moreover, one should consider poor prognostic factors such as organism type or host factors before the ultimate decision is made. Lastly, IPE of patellar components is most feasible with metal-backed components when significant wear is leading to osteolysis or patellar maltracking [71]. In the setting of axial malalignment and patellar maltracking without wear, IPE becomes less reliable. If performing an IPE on an all-PE patella component, beware of significant morbidity associated with extensor mechanism compromise.

One aspect not specifically mentioned within the review’s subsection is the potential cost savings associated with IPE compared to component revision. While cost is not necessarily considered in the management of any one particular patient, when considering population health, IPE has been shown to be significantly more cost-effective due to its reduced operative time, shorter length of stay, lower blood loss, and implant cost [73]. Of course, a failed IPE with subsequent component revision negates any cost savings, so proper surgical indication is critical. Limitations of current literature include the predominately retrospective, underpowered, and heterogenous definitions and outcomes in the available literature. There are very few prospective or randomized trials. Glaring gaps in the literature include large studies investigating prognostic factors, functional outcome measures, and subjective outcome measures comparing IPE with other known treatment options. Factors that we suggest being further studied include, but are not limited to, patient demographics, comorbidities, preoperative clinical severity, primary surgical complications, and preoperative pain. Additionally, although reoperation is a reasonable outcome measure for success, it must be acknowledged that patients and providers alike may be more willing to undergo a two-stage revision after IPE when compared with a second two-stage revision after an already failed two-stage revision. This makes re-revision as an outcome measure problematic in this context.

Future directions of study include the need for standardized definitions of success, whether that be survivorship, patient-reported outcome measures, or reoperations. Direct comparative cost-effectiveness studies may help with population management as our elderly population continues to grow. Newer data on improved implant designs and modern PE liners are needed, as they are promising in terms of being associated with reduced revision rates. Lastly, prediction strategies need to be improved. Studies evaluating positive or negative prognostic biomarkers may help evaluate who is best served with an IPE. Additionally, the use of AI prediction models may help evaluate which patients would benefit from IPE. 

## 5. Conclusions

IPE offers a bone-preserving and less morbid revision strategy in selected patients. Outcomes are best in well-fixed, well-aligned TKAs with favorable prognostic factors, depending on the particular indication for intervention. Poorer results occur in chronic infection, gross patellar maltracking, severe instability, or component loosening, where full component revision remains necessary. Evidence quality is low, and further prospective research is needed to establish definitive guidelines. Until then, IPE should be considered cautiously, tailoring the indication with patient factors, surgical considerations, and implant design options.

## Figures and Tables

**Table 2:** Instability.

Author	Year	Sample Size	Intervention	Follow Up	Outcome
Babis et al. [39]	2002	56	Isolated tibial insert exchange for wear/instability	Mean 4.6 years (range 2–14)	25% required re-revision; 5.5-year survivorship 63.5%; high early failure rate; modest improvement in Knee Society scores
Brooks et al. [40]	2002	14	Polyethylene exchange only for instability	Mean 56 months	71% stable knees; 29% failure rate; HSS Knee Score improved from 50 to 73
Willson et al. [41]	2010	42	Isolated tibial polyethylene insert exchange for instability, stiffness, or effusion	Mean 5.6 years (range 2–11)	29% required subsequent revision; 58% survivorship at 11 years; 30% persistent pain in unrevised patients
Cooper et al. [42]	2018	90	Isolated polyethylene exchange with increased constraint for instability	Mean 3.7 years (min 2 years)	ITPIE failure rate 19.4%; comparable outcomes to component revision; lower re-revision rate when constraint increased
Green et al. [43]	2020	31	Isolated polyethylene insert exchange for flexion instability	Mean 41 months (range 24–85)	6.5% required component revision; >90% success; significant improvement in pain and function scores
Tegethoff et al. [44]	2022	20	Isolated liner revision for instability	Not specified (retrospective, 2011–2018)	60% reoperation rate; 45% component revision; higher reoperation and infection rates vs. component revision

**Table 3:** Prosthetic Joint Infection.

Author	Year	Sample Size	Intervention	Follow Up	Outcome
Urish et al. [55]	2018	216	I&D with component retention	Median 14.3 months (up to 4 years)	57% failure at 4 years; 19.9% 5-year mortality; failure predicted by time symptomatic and organism; culture-negative status higher risk
Narayanan et al. [56]	2018	55	I&D with polyethylene liner exchange	Mean 2.5 years	Success 82% if I&D within 2 weeks of index TKA; 50% if >2 weeks; time from index TKA to I&D is key predictor
Son et al. [57]	2017	25	Open debridement + polyethylene exchange (strict selection: acute, well-fixed, no sinus tract)	Mean 2.5 years (range, 24 to 35 months).	Success 88%; strict selection (acute, no sinus tract, well fixed) critical for outcome
Zhang et al. [58]	2017	35	I&D with polyethylene insert exchange	Mean 4.5 years	Success 75%; insert exchange crucial for outcome; failure associated with delayed intervention
Estes et al. [59]	2010	20	Two-stage retention debridement protocol (repeat I&D, antibiotics, retention)	Mean 3.2 years (range 1.2–7.5 years)	Infection was controlled 90%; protocol may benefit select acute cases
Flierl et al. [60]	2017	32	I&D with antibiotic-impregnated calcium sulfate beads	Mean 12.7 months (range 3–30 months)	48% failure rate; poor outcomes with beads; high failure rate
Riesgo et al. [61]	2018	36 in VIP group	I&D with vancomycin-povidone-iodine protocol	Mean 2.2 years	83.3% success rate; protocol improved survivorship compared to historical controls
Hischebeth et al. [62]	2019	74	I&D, stratified by organism (MRSA/MSSA vs. MRSE/MSSE)	Mean 2.5 years	MRSA/MRSE associated with higher failure; organism type impacts outcome
Sherrell et al. [63]	2011	83	Two-stage reimplantation after failed I&D	Mean 4.2 years	Success 67% after two-stage; prior failed I&D worsens prognosis
Bene et al. [64]	2018	76	I&D with liner exchange	Mean 3.5 years	Success 72.4%; failure associated with symptom duration >7 days, comorbidities
Swenson et al. [65]	2018	71	Open debridement + polyethylene exchange	Mean 2.89 years (range 6 months–6.3 years)	26.4% failure rate; preoperative anemia associated with failure
Patel et al. [66]	2023	Review article; includes multiple studies (e.g., 34-patient cohort for synchronous PJI, large registry data for mortality)	Comprehensive review of PJI management (DAIR, one-stage, two-stage revision, suppressive antibiotics)	Variable (outcomes reported at 30 days, 1 year, 5 years, 10 years)	Appropriate for acute PJI with stable implants; less effective for chronic/late PJI; outcomes depend on timing, organism, and patient selection; two-stage revision preferred for chronic or complex cases
Wood et al. [67]	2025	660 (population based study)	tibial insert exchange (TIE), single-stage revision TKA (ssrTKA), and two-stage revision (tsrTKA)	2 years	19.2% re-revision rate with TIE, 27.9% with ssrTKA, 16.4% with tsrTKA; higher risk repeat revision with TIE (OR 1.77; 95% CI 1.12 to 2.81) and ssrTKA(OR 2.42; 95% CI 1.42 to 4.12); most for repeat infection; ssrTKA higher rate of repeat revision for persistent infection (OR 1.93, 95% CI 1.16–3.21)
